# Cases

**Published:** 1896-01

**Authors:** 

**Affiliations:** Kiel


### CASES.

By Dr. Kunkel of Kiel.

[From Die Allg. Hom. Zeitung. Translated by A. McNeil, M. D., San Francisco, Cal.]

G., æt. thirty-four, consulted me September 9th, 1893. He has suffered three years from asthma, which followed bronchitis. The attacks came more frequently at night and more particularly after becoming warm in a feather bed and also toward morning; during the day, when attending to his business, it is ameliorated. Aggravations by moist air. During childhood he was healthy, but with great disposition to catch cold, which caused a coryza, often profuse. He is now free from difficult respiration.

I gave him eight (8) powders of Sulphur$^{cc}$ (Lehrman's), one to be taken every seventh evening.

November 1st.—Owing to the distance at which he lived he could not report in person; he sent word: "I can say with praise and thanks to God that I have been free from asthma for weeks. I feel right well, better than ever before." Sulphur$^{300}$, a dose every fourteen days.

Anna K., æt. three, was brought to me November 16th, 1892. She belongs to a "psoric" family, whose physician I

have been for many years. Her present complaints are thread-worms, extreme irritability ; often she will not permit any one to approach her.

After a few doses of Cina$^{30}$, given at intervals of from five to seven days, in the evening, worms disappeared without a trace.

January 10th, 1893, she again returned. Since the last of December she has been afflicted with attacks of asthma, which come at night. She throws off all the bed-clothes, loss of appetite, aversion to milk, cold feet, blepharitis, diarrhœa occasionally, at different times of the day.

Sulphur$^{cc}$ (L.), for five or six days, then no medicine.

February 27th.—At first aggravation of diarrhœa, then stool became normal. Now a return of diarrhœa, twice at night; smells sour, and mixed with undigested food ; the feet which had become warm are now cold.

Sulphur$^{cc}$, five powders, one every 9th evening, since then she has remained well. I know, as I see her often.

The statement of Farrington that Cina is not suitable for thread-worms I cannot indorse.

Seh., farm laborer, æt. twenty, called January 14th, 1893. Well as a child, only that he was often the victim of boils. His feet sweat in summer, and are cold in winter. Sweats easily at his work and has short breath. He also often has nose-bleed. In the summer of 1892 sudden falling without premonition, and with loss of consciousness. After the attack headache and extreme prostration. He had an attack the middle of December and beginning of January. His companions had not observed any spasms—"epileptic vertigo." He had not perceived that the weather had any influence on him.

Sulphur$^{cc}$ (L.), six doses, one every week.

February 25th.—On the whole is better. The vertigo has not returned, nose-bleed twice.

The same medicine, a dose every ninth morning till six were taken. He felt well and was discharged with the necessary directions.

(The following is by Kunkel, *but is from Das Archiv. für Hom.*)

Mrs. P. consulted me January 13th, 1894. She was operated on in the beginning of August, 1893, in the left breast, to remove an induration. There were repeated relapses followed by operations the last time in February, but how often I did not learn. As far as the mammæ had been removed, an ulcerating surface, about twelve or thirteen centimetres in diameter (five or six inches), which showed no tendency to healing around the ulcer to a breadth of from five to eight centimetres (two to three inches); it was stone hard.

In the axillæ no trace of an enlargement. The patient is a fat blonde. She formerly suffered much from toothache, which was produced or aggravated by drafts or moisture. Menses profuse, too soon, leucorrhœa; formerly her hands sweat, particularly when working. Her husband is healthy, two children have died of "glandular disease."

Calcarea-carb.$^{cc}$ (L.), a dose every seventh evening.

July 24th.—At first there was profuse purulent discharge, now the ulcerated surface is smaller. The hardness of the surrounding tissue is almost gone.

September 10th.—Continued improvement. The neighborhood of the ulcer is of normal softness. The same remedy every ninth evening.

October 15th.—Since eight days the sore is healed. Has no complaints. Give four doses, one every fourteen days. (Why? Trans.).

Cases like this of induration of the cellular tissue, which always relapse after an operation, do not often occur. We cannot properly think of cancer.

Therapeutically, the question arises, what remedy should have been given in accord with the principles of the physiological school, and what in that of (our) materia medica? In regard to the former it might occur to an antagonist: *quot capita tot sensus*, the answer; to the latter the reply would be tolerably unanimous.

Miss P., æt. twenty years, consulted me May 1st, 1893. She had suffered from gastric trouble for four years. She has sometimes, without intermission, pains in the stomach all day (nights free), and sometimes coming in paroxysms. The pain is pressing, comes after eating or is aggravated thereby sometimes immediately, and other times a half hour after, constipation; leucorrhœa before menses; slight sweat on motion, particularly on the head; feet cold; epigastrium swollen, requiring her to unhook her dress; vomiting of the ingesta, of acids, and sour water; in winter this vomiting is aggravated.

Calc.$^{cc}$ (L.), a dose every seventh evening.

July 3d.—Improvement of her general condition, but is not yet well. Has some pain in stomach and occasionally vomiting. The same medicine.

September 9th.—After the first three powders "quite beautiful," but is now worse; extremely cold feet and cold sweat on them; vomiting of the ingesta immediately after eating; flatulence, particularly in the afternoon.

Lycopod.$^{cc}$ (L.), eight doses, one every seventh evening; when all taken, patient felt well.

E., teacher, æt. thirty-two years; formerly healthy, only that he often suffered from toothache, which was caused or aggravated by a draft; consulted me September 1st, 1894. He has suffered from four to five years from headache, usually on one side, but at times in the entire head. It comes at longer or shorter intervals, usually in the forenoon, becoming worse in the afternoon till evening. Slight sweat on movement, particularly on the head and face; vertigo, especially on stooping. Aggravated by washing himself and during moist, cloudy weather. Aversion to physical as well as mental labor. The ability to think has suffered.

Calc-c.$^{200}$ (L.), six doses, one every week.

October 27th.—Essential improvement, of the headache there is only a trace; disposition decidedly better; desire for mental and physical exercise; "ability to think better." He did not return until March 3d, 1895. Lately the headache has reappeared, but mental work is done easily and memory is better. The same medicine, a dose every two weeks.

Fr., æt. thirty-three years, consulted me March 14th, 1893. He says that he has suffered ten or twelve years from gastric pains. Before that nose-bleed and toothache, which was aggravated by drafts. The pain in stomach is pressing, is not aggravated by eating, comes about six P. M. and before midnight, with the pains distention of the epigastrium ; lying on abdomen ameliorates.

Calc-c.$^{200}$, six powders, one to be taken every seventh evening.

December 15th, 1893, he returned. He has in the interval been quite well, no pressure in the stomach. It reappeared three weeks ago. Momentary improvement after eating, aggravation at 5 P. M. and late in the evening. There is again distention of the epigastrium with the pains, and also amelioration when lying on abdomen.

Calc-c.$^{200}$, a dose every seventh evening.

January 19th, 1894.—No improvement the first week, since then free from pain ; no lying on abdomen. I again ordered Calc-c.$^{200}$, a dose every ninth evening, which completed the cure.

